# Effects of Interspecific Competition on Habitat Shifts of *Sardinops melanostictus* (Temminck et Schlegel, 1846) and *Scomber japonicus* (Houttuyn, 1782) in the Northwest Pacific

**DOI:** 10.3390/biology14080968

**Published:** 2025-08-01

**Authors:** Siyuan Liu, Hanji Zhu, Jianhua Wang, Famou Zhang, Shengmao Zhang, Heng Zhang

**Affiliations:** 1 Key Laboratory of Oceanic and Polar Fisheries, Ministry of Agriculture and Rural Affairs, P.R. China, East China Sea Fisheries Research Institute, Chinese Academy of Fishery Sciences, Shanghai 200090, China; 18336156726@163.com (S.L.); mikey0987@163.com (H.Z.); wjh20001231@163.com (J.W.); 13276209271@163.com (F.Z.); ryshengmao@126.com (S.Z.); 2 College of Marine Living Resource Sciences and Management, Shanghai Ocean University, Shanghai 201306, China; 3 College of Navigation and Ship Engineering, Dalian Ocean University, Dalian 116023, China

**Keywords:** *Sardinops melanostictus*, *Scomber japonicus*, the northwest Pacific, interspecific relationship, ecological modeling

## Abstract

As economically important sympatric species in the Northwest Pacific, the Japanese sardine (*Sardinops melanostictus*) and Chub mackerel (*Scomber japonicus*) exhibit significant biological interactions. Understanding the impact of interspecies competition on their habitat dynamics can provide crucial insights for the sustainable development and management of these interconnected species resources. This study utilizes fisheries data of *S. melanostictus* and *S. japonicus* from the Northwest Pacific, collected from June to November between 2017 and 2020. We integrated various environmental parameters, including temperature at different depths (0, 50, 100, 150, and 200 m), eddy kinetic energy (EKE), sea surface height (SSH), chlorophyll-a concentration (Chl-a), and the oceanic Niño index (ONI), to construct interspecific competition species distribution model (icSDM) for both species. We validated these models by overlaying the predicted habitats with fisheries data from 2021 and performing cross-validation to assess the models’ reliability. Furthermore, we conducted correlation analyses of the habitats of these two species to evaluate the impact of interspecies relationships on their habitat dynamics. The results indicate that, compared to single-species habitat models, the interspecific competition species distribution model (icSDM) for these two species exhibit a significantly higher explanatory power, with *R*^2^ values increasing by up to 0.29; interspecific competition significantly influences the habitat dynamics of *S. melanostictus* and *S. japonicus*, strengthening the correlation between their habitat changes. This relationship exhibits a positive correlation at specific stages, with the highest correlations observed in June, July, and October, at 0.81, 0.80, and 0.88, respectively; interspecific competition also demonstrates stage-specific differences in its impact on the habitat dynamics of *S. melanostictus* and *S. japonicus*, with the most pronounced differences occurring in August and November. Compared to *S. melanostictus*, interspecific competition is more beneficial for the expansion of the optimal habitat (HIS ≥ 0.6) for *S. japonicus* and, to some extent, inhibits the habitat expansion of *S. melanostictus*. The variation in migratory routes and predatory interactions (with larger individuals of *S. japonicus* preying on smaller individuals of *S. melanostictus*) likely constitutes the primary factors contributing to these observed differences.

## Simple Summary

Japanese sardine (*Sardinops melanostictus*) and Chub mackerel (*Scomber japonicus*) in the Northwest Pacific have important biological interactions. This study uses 2017–2020 fisheries data and environmental parameters to build interspecific competition species distribution model (icSDM). Validation with 2021 data demonstrates that icSDM exhibit greater explanatory power compared to single-species suitable habitat model (ssSDM). Interspecific competition influences the habitat dynamics of the two species, enhancing the correlation in habitat changes, particularly in June, July, and October. There are stage-specific differences, with August and November showing the most. Competition benefits *S. japonicus*’ optimal habitat expansion more than *S. melanostictus*’, likely due to migratory and predatory differences.

## 1. Introduction

Climate and environmental changes have altered the original distribution of marine species, resulting in the displacement of their suitable habitats as environmental conditions change [1]. In the northwestern Pacific, mesopelagic and epipelagic fish species exhibit substantial similarity in prey composition, along with interspecific biological interactions, which lead to a high degree of overlap and interlacing in the distribution of their optimal habitats [2]. Japanese sardine (*S. melanostictus*) and Chub mackerel (*S. japonicus*) are two major economic fish species in the Northwest Pacific, with highly similar habitat distributions. Moreover, environmental changes lead to significant shifts in their habitats. Research has found that the fishing grounds of *S. melanostictus* and *S. japonicus*, along with their Habitat Suitability Index (HSI), shift in response to environmental fluctuations. Seasonal distribution shifts were consistent, with northeastward movements in spring and autumn, and southwestward shifts in autumn and winter [3,4]. Additionally, according to statistical data from the North Pacific Fisheries Commission (NPFC), over the past decade, both *S. melanostictus* and *S. japonicus* have ranked among the top catches in the Northwest Pacific. Their annual production shows alternating trends. In 2018, for the first time in nearly 20 years, the annual production of Japanese sardine exceeded that of Chub mackerel, and this gap continues to widen [5]. The variability in small-scale environmental factors is the direct cause of annual differences in fishing grounds and habitats. Meanwhile, the large-scale climate fluctuations constitute the underlying factors responsible for the significant decadal-scale alternations in the resources of these two fish species [6,7]. Therefore, understanding the patterns of habitat suitability changes and the dynamic variations in habitat distribution of both species is crucial for the sustainable utilization and management of fisheries resources.

Currently, there has been extensive research on the suitable habitat distributions of *S. melanostictus* and *S. japonicus* in the Northwest Pacific [8,9,10]. Most studies have utilized Species Distribution Models (SDM) [11] to examine the relationship between environmental variables and species distribution. Since 2019, convolutional neural networks have been employed to identify the distribution of optimal habitat centers for *S. japonicus* within the primary fishing grounds (39° N–43° N, 149° E–154° E) [12]. A concentration distribution model reveals that *S. melanostictus* migrates northeast in June and southwest in August [9]. MaxEnt modeling of *S. melanostictus* and *S. japonicus* indicates that both species share similar optimal habitats during summer (39° N–42° N), and in autumn *S. melanostictus* concentrates between 39° N and 44° N, while *S. japonicus* is found between 40° N and 43° N. Key environmental factors influencing habitat dynamics include sea surface height (SSH), sea surface temperature (SST), and chlorophyll-a (*Chl.a*) [13]. Additionally, the classification tree model indicates that the habitat range of *S. melanostictus* is broader in June, expanding from September to November, while the suitable habitat area for *S. japonicus* decreases over time [12,14]. Previous studies have shown that, during the juvenile stage, *S. melanostictus* and *S. japonicus* occupy comparable trophic positions within the food web and exhibit highly similar feeding spectra, primarily composed of copepods and small shrimp species, suggesting a clear food-driven interspecific competition [10,11,12,13,14]. As they mature, *S. japonicus*, owing to its larger body size, expands its diet to include small fish, thereby reducing the intensity of direct competition with *S. melanostictus*. However, crustaceans and cephalopods remain the major dietary components for both species, indicating that trophic overlap and food-mediated competitive interactions persist even at the adult stage. Comparing multiple studies reveals a significant correlation in the habitat dynamics between *S. melanostictus* and *S. japonicus*. When their distribution positions are close, it promotes suitable habitat conditions for both species. Conversely, when their distribution positions are farther apart, it tends to inhibit the increase in habitat area for *S. japonicus* [15,16,17,18]. This suggests that the changes in suitable habitats for both species may be influenced by certain underlying non-biological environmental factors, causing the habitats of *S. melanostictus* or *S. japonicus* to change in response to changes in the habitats of associated species [19].

Many methods within Ecological Niche Modeling (ENM) [3] and Species Distribution Modeling (SDM)—including algorithms such as the Generalized Additive Model (GAM) [20], Habitat Suitability Index (HSI) [21], and Random Forest (RF) [22]—are used to identify the optimal non-biological environmental conditions for individual species. These approaches integrate variations in environmental factors with changes in suitable habitats, enabling the modeling of species’ habitat distributions. Integrating environmental data with fishery data offers a scientifically robust approach to identifying and predicting the optimal habitat distribution of individual species. The Random Forest (RF) method, in particular, provides a reliable and effective means for such habitat suitability modeling. Notably, the prediction performance of the Random Forest model has shown high accuracy, high explanatory power, and a high weight ratio across various fish species (such as *S. japonicus* [23,24], *Thunnus alalunga* (Bonnaterre, 1788) [25], etc.) However, modeling based solely on the relationship between non-biological environmental factors and yield lacks comprehensiveness and objectivity for studying habitat changes of associated species (such as *S. melanostictus* and *S. japonicus*). This approach overly emphasizes the response of habitat changes to non-biological factors while underestimating the dynamic correlation of biological interactions between the species [13,26], such as the impact of interspecies competition and predation relationships on habitat changes. In addition, a comprehensive analysis of 688 published studies revealed significant variability in the impacts of global change drivers on interactions among any type of organisms, both in terms of the magnitude of changes and the degree of directional shifts [27]. Interactions among organisms can influence species responses to non-biological environmental changes along environmental gradients. Similarly, changes in non-biological environments also affect the nature of biological interactions [28,29]. Therefore, it can be argued that the extent to which climate and environmental changes at different scales affect species habitat distribution is constrained by interactions among species. Ignoring these interactions would reduce the accuracy and comprehensiveness of predictions regarding species habitat distribution [11].

Therefore, leveraging fishery data and marine environmental data, this study employs the Habitat Suitability Index to characterize the biological competition between *S. melanostictus* and *S. japonicus*. By integrating medium- and large-scale environmental and climatic factors with competitive interactions between these species and embedding the biological parameters of *S. melanostictus* and *S. japonicus* into a Random Forest model, we develop a dynamic interspecific habitat model. This model aims to elucidate the cohabitation habitat dynamics of the species in the Northwest Pacific high seas, assess the correlation of their habitat distributions, and analyze the impact of interspecific competition on the correlation and divergence of their habitat distributions. The results provide a scientific basis for the rational development and management of these two pelagic species population resources.

## 2. Materials and Methods

### 2.1. Data Sources

#### 2.1.1. Fisheries Data

The fisheries data in this study were sourced from fishing logs recorded by Chinese commercial light purse seine vessels. The study area mainly covers the range of 34° N–45° N and 144° E–163° E, and the temporal scope of this study spans the primary fishing season between June and November from 2017 to 2021. The date, latitude, longitude, catch (measured in tons), hauls, vessel length, and vessel ID were included in the logbook data, and the location data was grouped by 0.25° × 0.25° spatial extent. The sample sizes for modeling and validation of *S. melanostictus* and *S. japonicus* are shown in Table 1.

Each month, 30 individuals of *S. melanostictus* and *S. japonicus* were randomly sampled. Fork length and body weight were recorded for each specimen, and stomach content analysis was performed to characterize dietary composition and evaluate interspecific differences in feeding habits and growth patterns across months.

#### 2.1.2. Environmental Data

Previous research has demonstrated that SSH, Chl-a, and SSS exert significant influence on the abundance and spatiotemporal distribution of *S. melanostictus* and *S. japonicus* populations [3,4,5]. Given that both species belong to small-to-medium-sized pelagic fish, distributed within the 0–200 m water column [30], different temperature gradients across various water depths (0 m, 50 m, 100 m, 150 m, and 200 m) were selected for analysis. Furthermore, both species are primarily distributed in the convergence zones of the Kuroshio Current and its associated subcurrents, where mesoscale eddies, oceanic fronts, and meanders of the Kuroshio extension can influence the migration of small-to-medium-sized pelagic fish [16]. Therefore, this study supplements environmental factors such as eddy kinetic energy (EKE) and El Niño-Southern Oscillation (ENSO, represented by the oceanic Niño index, ONI). Environmental data processing and map design in this study were conducted using ArcGIS 10.8 and MATLAB R2021b software. The spatial resolution and sources of environmental data are illustrated in Table 2:

### 2.2. Methods of Analysis

#### 2.2.1. Constructing Single-Species Suitable Habitat Models (Single-Species SDM, ssSDM)

**Figure 1:**
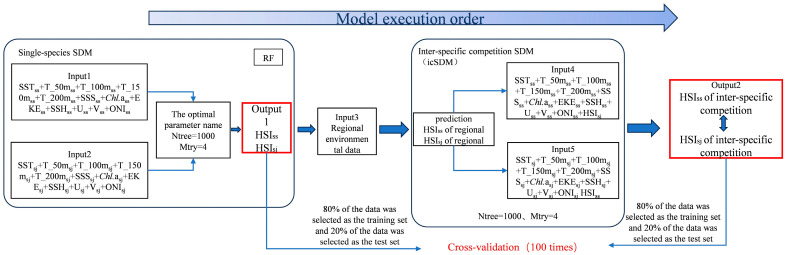
The process for constructing interspecific competition species distribution models for *S. melanostictus* and *S. japonicus*.

The HSI is employed to quantitatively characterize the suitability of growth conditions within a species’ distribution range. It enables a quantitative evaluation of the extent to which environmental factors affect the species in a specified area [31]. Fishing effort serves as a metric for validating model accuracy, effectively mitigating errors introduced by fishing gear, techniques, and anthropogenic factors [32]. Based on findings from previous research on the distribution of pelagic fish [23,25,33], the RF model stands out as the most effective SDM for fitting species distribution patterns. Optimal RF model performance is achieved with ntree set to 1000 and mtry set to 4. Therefore, this study employs the Random Forest model to construct single-species habitats for *S. melanostictus* and *S. japonicus*, with fishing effort used as a metric to validate the effectiveness of the model construction. The environmental data of *S. melanostictus* and *S. japonicus* in the Northwest Pacific from June to November 2017–2020 were matched with corresponding fishing data at specific sites. These matched fishery–environmental datasets were then inputted into a Random Forest model with ntree = 1000 and mtry = 4 to optimize the weighting of various environmental factors. Subsequently, optimal habitats, denoted as HSIss for *S. melanostictus* and HSIsj for *S. japonicus*, were constructed based on the model outputs. The procedural details are depicted in Figure 1.

#### 2.2.2. Construction of the Interspecific Competition Species Distribution Model (icSDM)

Biological interactions can variably influence species responses to non-biological environmental changes along environmental gradients, thereby impacting species habitat dynamics [11,28,29]. Therefore, this study utilized environmental data from June to November during the years 2017 to 2020 in the Northwest Pacific region (145° E–163° E, 34° N–45° N) to input into the optimal models HSIss and HSIsj. These models were used to estimate the habitat suitability indices for *S. melanostictus* and *S. japonicus* across the entire study area, referred to as HSIss of regional and HSIsj of regional, respectively. These suitability indices were subsequently incorporated as biotic factors into the habitat suitability models for both species, enabling the construction of an interspecific relationship habitat model. This model was then employed to predict the suitable habitats for *S. melanostictus* and *S. japonicus* for the period from June to November 2021, referred to as HSIss of interspecific competition and HSIsj of interspecific competition, respectively. Incorporating the effort data of *S. melanostictus* and *S. japonicus* from June to November 2021 with the predicted suitable habitats derived from the interspecies relationship habitat models, HSIss, HSIsj, HSIss of interspecific competition, and HSIsj of interspecific competition were partitioned into training (80%) and testing (20%) datasets. This partitioning was performed for 100 iterations of leave-one-out cross-validation to assess the reliability of the icSDM for predicting the effectiveness of habitats for *S. melanostictus* and *S. japonicus* [23,25,33]. Details of this process are illustrated in Figure 1.

#### 2.2.3. Building on the Framework of Interspecific Competition, the Temporal Variations in Suitable Habitats for *Sardinops melanostictus* and *S. japonicus* Were Analyzed Both on a Monthly and Annual Scale

This study partitioned the HSI, ranging from 0 to 1, for cohabitation of *S. melanostictus* and *S. japonicus* during June to November 2021. Based on the species’ suitability, the indices were classified into five intervals: 0 ≤ HIS < 0.2, 0.2 ≤ HSI < 0.4, 0.4 ≤ HSI < 0.6, 0.6 ≤ HSI < 0.8, and 0.8 ≤ HSI ≤ 1. Areas where HSI ≥ 0.6 were identified as the optimal habitats for both species [34,35]. Distribution maps of potential habitats for *S. melanostictus* and *S. japonicus* during June to November 2021, as well as for the period spanning 2017 to 2021, were generated using MATLAB software [7].

## 3. Results

### 3.1. Development of the icSDM for S. melanostictus and S. japonicus

**Figure 2:**
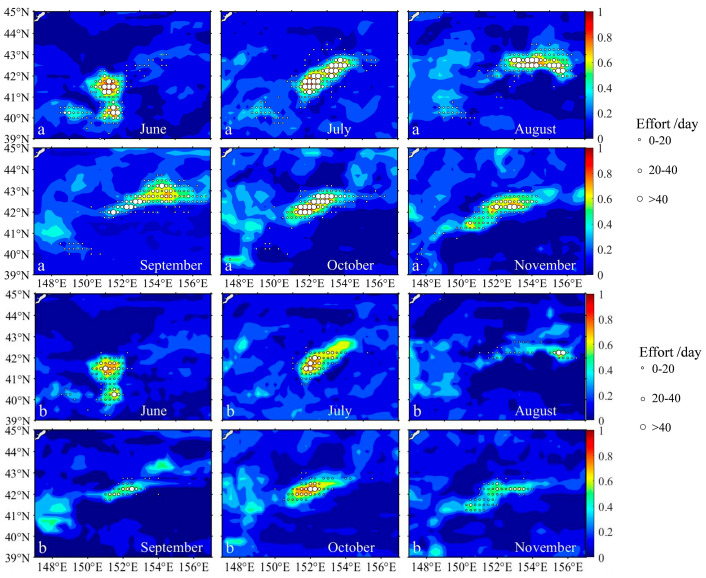
The predicted Habitat Suitability Index by joint interspecific competition species distribution model (icSDM) overlain with fishing efforts of *S. melanostictus* (**a**) and *S. japonicus* (**b**) from June to November in 2021. Note: “Effort” refers to the total number of fishing days undertaken by all operating fishing vessels at the corresponding site. “a” represents *S. melanostictus*. “b” represents *S. japonicus*.

Validation of the icSDM model developed in Section 2.2.2 was conducted using fisheries data of *S. melanostictus* and *S. japonicus* from June to November 2021. The resulting habitat distributions for both species are illustrated in Figure 2. The results showed that from June to November, areas with moderate fishing effort (20 day ≤ Effort < 40 days) for *S. melanostictus* and *S. japonicus* were predominantly concentrated in regions where HSI ≥ 0.6. Locations with the highest fishing effort (Effort ≥ 40 days) were generally in areas with the maximum HSI values. For *S. melanostictus,* 84% of the total fishing effort was in optimal habitats (HSI ≥ 0.6), while for *S. japonicus*, 78% of the total fishing effort was in these optimal habitats. Overall, the yield and fishing effort for *S. melanostictus* and *S. japonicus* were proportional to the HSI values across different HSI intervals. Thus, the icSDM models developed in this study for *S. melanostictus* and *S. japonicus* demonstrate a high capacity for accurately assessing and predicting their habitat conditions.

### 3.2. Validation of the Impact of Interspecific Competition on the Habitat Distribution of Both Species

**Figure 3:**
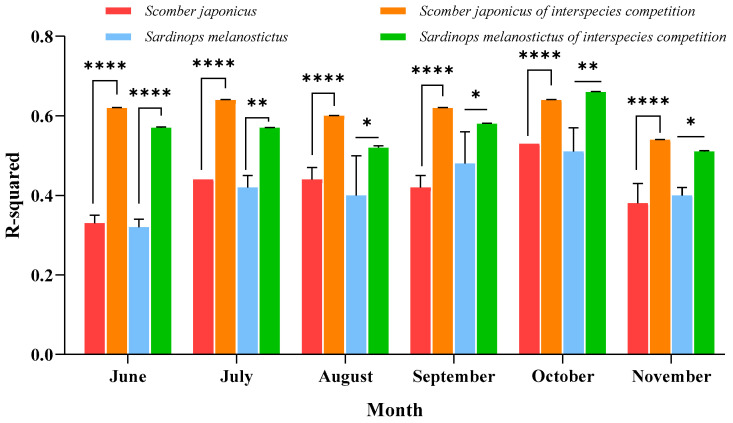
Fitting accuracy of ssSDMs and icSDMs models from June to November. Fitting accuracy of the ssSDM and icSDMs models for *S. melanostictus* and *S. japonicus* from June to November. Note: Different symbols above the bars denote the significant difference among groups at the same time point (* *p* < 0.05, ** *p* < 0.01, **** *p* < 0.0001).

Cross-validation of the interspecific competition habitat dynamic model (interspecific competition SDM, icSDM) was conducted and compared with the single-species SDM (ssSDM) models for *S. melanostictus* and *S. japonicus*. The results, presented in Figure 3, revealed that incorporating the habitat suitability of a single species (*S. melanostictus* or *S. japonicus*) as a biotic indicator to quantify interspecific competition into the habitat distribution model of the associated species (*S. melanostictus* and *S. japonicus*) significantly enhances the model’s fitting accuracy for both species. For *S. melanostictus*, the incorporation of *S. japonicus* distribution factors enhanced the explanatory power (R^2^) of the cross-validated models for each month, with increases ranging from 0.09 to 0.26. For *S. japonicus*, the inclusion of *S. melanostictus* distribution factors resulted in an improvement in the explanatory power (R^2^) of the monthly fitted models, with increases ranging from 0.11 to 0.29. In summary, the interspecific competition between *S. melanostictus* and *S. japonicus* significantly influences their habitat distribution.

### 3.3. The Impact of Interspecific Competition on Habitat Dynamics in S. melanostictus and S. japonicus

**Figure 4:**
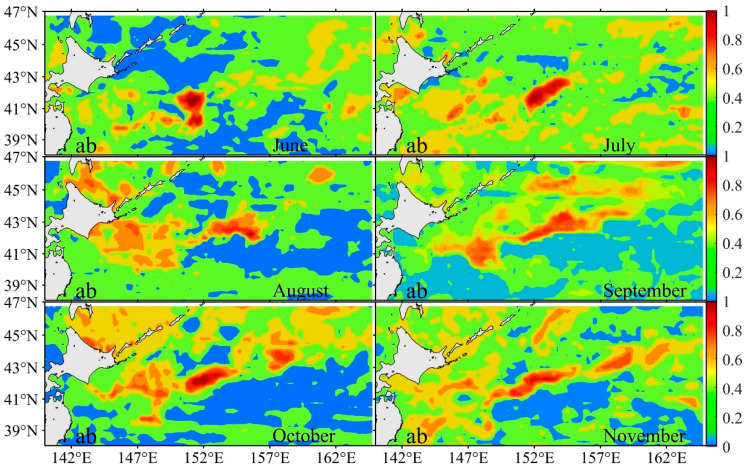
The syntopic distributions habitat of *S. melanostictus* (a) and *S. japonicus* (b) from 2017 to 2021.

The cohabitation habitat maps of *S. melanostictus* and *S. japonicus* from June to November 2021 are depicted in Figure 4. Overall, the cohabitation habitats of *S. melanostictus* and *S. japonicus* are primarily influenced by the distribution factors of *S. melanostictus*. Comparing the habitat distributions of both species (Figure 2) with their cohabitation habitats (Figure 4), monthly variations indicate minimal differences in habitat distribution during June, July, and October. The suitable habitats are predominantly concentrated in the region spanning 151° E–153° E and 40° N–43° N, where habitats with HSI ≥ 0.8 are consistently present. In August, September, and November, the cohabitation habitats of *S. melanostictus* and *S. japonicus* (Figure 4) show greater similarity with the suitable habitat distribution of *S. melanostictus* but exhibit notable differences from that of *S. japonicus*. The most significant disparities are evident in August and November, where *S. japonicus*’s suitable habitats are confined mainly to the area between 155° E–156° E and 41.5° N–42.5° N. In contrast, cohabitation habitats are distributed more broadly across the seas spanning 152.5° E–157° E and 41° N–43° N during these months. By November, the distribution of *S. japonicus* habitats (HSI ≥ 0.6) appears narrower compared to the cohabitation habitat distribution. Overall, the variations in optimal habitats for *S. melanostictus* and *S. japonicus* exhibit differential responses to factors influencing interspecific competition. The former demonstrates a positive correlation, whereas the latter demonstrates a negative correlation.

### 3.4. The Correlation Between Habitat Variations of Sardinops melanostictus and S. japonicus

**Figure 5:**
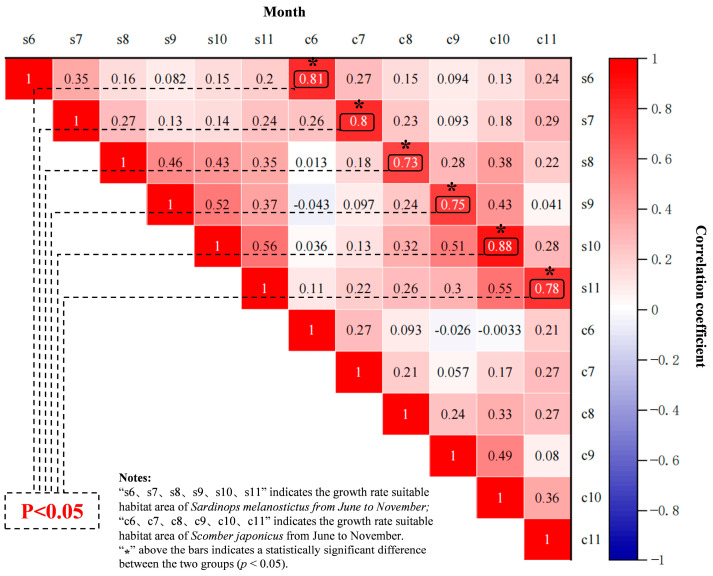
Correlation between the habitat (HSI) of *S. melanostictus* and *S. japonicus* from June to November in 2021.

Correlation analysis was conducted on the HSI of *S. melanostictus* in the Northwest Pacific region (145° E–163° E, 34° N–45° N) from June to November 2021 (Figure 5, s6, s7, s8, s9, s10, s11) and the corresponding HSI of *S. japonicus* in the same region (c6, c7, c8, c9, c10, c11). The results, as depicted in Figure 5, indicate a positive correlation between the predicted suitable habitats of *S. melanostictus* and *S. japonicus* incorporating interspecies competitive factors. In June, July, August, September, October, and November, there was a significant positive correlation (*p* < 0.05) in the distribution of suitable habitats between *S. melanostictus* and *S. japonicus*. Notably, the highest correlation was observed in October, suggesting that substantial changes in the habitat distribution of either *S. melanostictus* or *S. japonicus* are accompanied by corresponding shifts in the habitat of the associated species. The monthly changes in habitat distribution for *S. melanostictus* exhibit a weak positive correlation (maximum coefficient of 0.46). In contrast, the habitat distribution of *S. japonicus* shows a positive correlation across months, except for October and November (with a correlation coefficient of 0.49), which are generally lower than those observed for *S. melanostictus*. Additionally, the highest degrees of correlation in habitat distribution between the two species occurred in June (0.81), July (0.80), and October (0.88). In summary, there is a significant positive correlation in the distribution of suitable habitats for both *S. melanostictus* and *S. japonicus*. The interspecific competition between them is identified as one of the primary reasons contributing to this phenomenon.

### 3.5. Fork Length, Body Weight, and Stomach Content Composition of Sardinops melanostictus and Scomber japonicus

**Figure 6:**
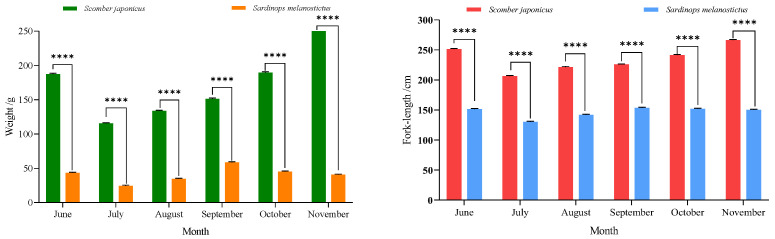
Monthly changes in the mean fork length and weight of *S. melanostictus* and *S. japonicus* from June to November 2021. Note: different symbols above the bars denote the significant difference among groups at the same time point (**** *p* < 0.0001).

**Figure 7:**
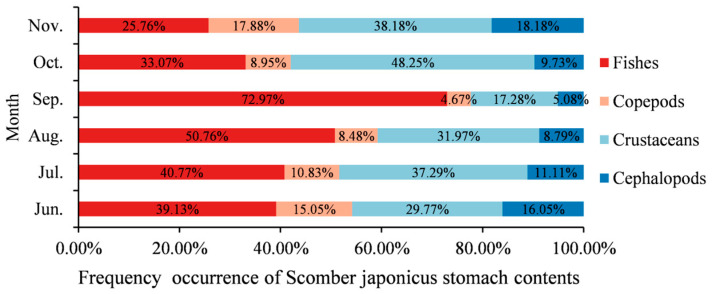
Monthly changes in the composition of stomach contents of *S. japonicus*.

In order to better understand the monthly differences in catch size, body weight, and feeding characteristics between the two populations, we analyzed information such as fork length, body weight, and bait composition from June to November. From 2017 to 2021, significant differences were observed in fork length and body weight between Japanese sardine and chub mackerel every year (Figure 6). The body weight and fork length of *S. japonicus* were consistently greater than those of *S. melanostictus* across all months, with the disparity generally increasing as the months advanced. Analysis of the stomach contents of *S. japonicus* indicated that the average proportion of fish prey was 0.44 across months, with a maximum value of 0.73 (Figure 7).

## 4. Discussion

### 4.1. Advantage Analysis of Establishing an icSDM

In the Northwest Pacific, the primary fishing grounds for *S. melanostictus* and *S. japonicus* are situated at the confluence of the Kuroshio warm current and the Oyashio cold current, characterized by complex water mass structures. The migratory behaviors and ecological habits of these two species exhibit a consistent response to environmental variations in this region, resulting in partial overlap of their fishing grounds [2,3,4]. Previous research has fitted the correlation between the fishing grounds or habitats of *S. melanostictus* and *S. japonicus*. For example, predictions using the maximum entropy model indicate that the potential cohabitation distribution of *S. melanostictus* and *S. japonicus* spans from 147° E to 157° E and 39° N to 44° N [2]. The optimal habitat model for *S. melanostictus* and *S. japonicus*, constructed using the arithmetic weighting method, demonstrates a significant dynamic relationship between their habitats [12]. Dai et al. (2017) [36] utilized GAM to fit the fishing ground characteristics of *S. japonicus* in the Northwest Pacific, identifying 39° N–43° N, 147° E–154° E as the primary fishing grounds for this species; Shi et al. (2023) [4] employed an integrated model to fit the potential fishing grounds of *S. melanostictus*, predicting a clear correspondence between the habitat and fishing grounds of this species. The outcomes of the aforementioned models demonstrate a relative alignment between predicted and observed values, consistent with the HSI ≥ 0.6 criterion typical of high-productivity zones. However, delineating clearly non-potential habitats (HSI ≥ 0.2) remains ambiguous. Moreover, the projected distribution range of optimal habitats notably exceeds that of actual fishing grounds. In this study, we assigned appropriate weights to interspecies competitive relationships between *S. melanostictus* and *S. japonicus*, utilizing a dynamic interspecies habitat model to delineate potential habitats. Our findings (Figure 1) reveal a notable spatial correspondence between the optimally fitted habitat regions and actual areas. The prediction accuracy for high-productivity zones (Effort ≥ 40 days) reaches 95%, with areas exhibiting minimal human fishing effort typically showing HSI values below 0.2 (Figure 1). Furthermore, our predictions demonstrate continuity without significant fragmentation, consistent with established patterns of species’ optimal habitat distribution [4]. Therefore, integrating interspecies biological interactions is inferred to be a crucial factor contributing to enhanced predictive model performance. Hence, this study comprehensively integrates environmental factors influencing habitat changes for *S. melanostictus* and *S. japonicus*, as well as interspecies competitive relationships between them, to construct an icSDM (Figure 1) that enhances both comprehensiveness and scientific rigor.

### 4.2. The Impact of Interspecies Competitive Relationships Between S. melanostictus and S. japonicus on Habitat Changes

#### 4.2.1. The Significance of Interspecies Competitive Relationships in Driving Habitat Changes Among Associated Species

As small-to-medium-sized pelagic fish, *S. melanostictus* and *S. japonicus* share common responses to environmental changes, exhibiting similar habitat distribution areas. They occupy comparable trophic levels in the food chain, feeding on copepods and small shrimp, which underscores a food-driven biological competition between them [37,38]. The cross-validation results of the icSDM in this study, compared with single-species optimal habitat results, visually demonstrate the significant impact of interspecies competitive relationships on the habitat distribution of *S. melanostictus* and *S. japonicus*. This indicates that biological relationships between associated species directly influence their habitat distribution (Figure 3). Akia et al. (2021) [26] and Liu et al. (2023) [8], investigating the habitat of *Scomberomorus niphonius* in China’s coastal waters, incorporated the habitat distribution of the primary prey species *S. japonicus*. They observed that the northward shift in *S. japonicus* habitats is a key factor contributing to the annual contraction of S. niphonius habitats. Fuji et al. (2023) [2] investigated the distribution patterns of small pelagic fish in the Northwest Pacific, revealing that interspecific competition is the primary driver behind the staggered distribution changes observed in Cololabis saira, *S. melanostictus*, *S. japonicus*, and *Engraulis japonicus*. The study highlights that fluctuations in the habitat of one species induce corresponding shifts in the habitats of competing species, indicating a cascading effect in their spatial dynamics [39,40]. Habitat changes inevitably result in alterations in biomass. Over the past 30 years, the populations of *S. melanostictus* and *S. japonicus* have exhibited significant alternations. Yatsu (2019) [41] attributed these long-term dynamics primarily to large-scale climate oscillations, with short-term variations linked to fluctuations in SST. However, we propose that interspecific competition between these two species provides a complementary explanation for the observed short-term alternations in their populations. In summary, for *S. melanostictus* and *S. japonicus*, interspecific competition has intensified the correlation between their habitat changes.

#### 4.2.2. Divergent Responses of Interspecific Competition to Habitat Changes for Both Species

The population sizes of *S. melanostictus* and *S. japonicus* are largely contingent upon recruitment rates, which are in turn governed by habitat environmental conditions. Within the framework of specific metabolic efficiencies and maternal effects, the influence of interspecific factors on habitat changes exhibits considerable variability and complexity [14,17,19,42]. The distribution of the cohabitation habitats of S.melanostictus and *S. japonicus* in August, September, and November (Figure 4) closely aligns with the distribution of suitable habitats for *S. melanostictus* (Figure 2) but differs markedly from those of *S. japonicus* (Figure 2). Furthermore, the yield changes between August and September exhibit slight variations, with *S. melanostictus* showing a decrease and *S. japonicus* an increase. Kamimura et al. (2021) [42] observed annual variations in *S. japonicus* resources, noting a trend where increased abundance of *S. melanostictus* coincided with decreased abundance of *S. japonicus*. This pattern reflects the alternating resource dynamics driven by interspecific competition between these species. Furthermore, research has revealed that from August to October, the fishing grounds of *S. melanostictus* and *S. japonicus* reach the easternmost part of the sea and subsequently begin a westward retreat. Notably, the centroid of *S. melanostictus*’ fishing grounds extends to 154.5° E, whereas that of *S. japonicus* reaches only 153° E [4,19,24]. The observed phenomena are likely due to the superior migratory abilities of *S. melanostictus*, which enable it to access more distant marine areas and expand its distribution eastward. In contrast, *S. japonicus*, influenced by the long-distance migrations of associated species, experiences reduced competition within cohabitation habitats, resulting in a temporary increase in productivity [6,8]. In addition, constrained by regional food availability and predation dynamics where larger *S. japonicus* prey on smaller *S. melanostictus*, habitats of *S. japonicus* expand concomitantly with the spread of fish prey (*S. melanostictus*) when crustacean densities decline in the area [8,43,44]. Thus, it is hypothesized that as cohabitation habitats enlarge alongside *S. japonicus* habitats, the latter’s presence increases due to heightened interspecific competition, thereby restraining the expansion of *S. melanostictus* habitats. During winter, warm temperate epipelagic fish migrate northward for overwintering and enter spawning grounds. Both *S. melanostictus* and *S. japonicus* move southwestward during this period. As they search for suitable spawning grounds, competition for food diminishes, leading to temporarily similar habitat changes [45,46,47]. This also corroborates our findings that, in November, the changes in cohabitation habitats and single-species habitats for both species exhibit consistent patterns. In summary, the migratory routes and seasonal spawning behaviors of *S. melanostictus* and *S. japonicus* are pivotal in driving the differential impacts of interspecific competition on their habitat changes.

#### 4.2.3. The Effect of Predation and Being Preyed upon on the Interspecific Competitive Relationship Between the Two Species

Both *S. japonicus* and *S. japonicus* are schooling fish species that exhibit medium-to-long-distance migratory behavior, with overlapping feeding grounds and migration routes. Analysis of their mean fork length and body weight (Figure 6) revealed that *S. japonicus* consistently showed significantly greater body weight and fork length than Japanese sardine across all sampled months, with this size disparity progressively increasing with the arrival of autumn and winter (August-November). Analysis of the stomach contents and trophic level of *S. japonicus* revealed that the relative importance index (*IRI*) of *S. melanostictus* as prey was 12.34, with a weight percentage (w) of 36.04. (Tang et al., 2020 [48]). Individual size also significantly influences the trophic dynamics between the two species. In the stomach of large mackerel, we often find complete or undigested sardine, which proves that larger mackerel (probably above 20% individuals) will directly prey on sardines, especially in the autumn–winter (lately August-November) (Figure 7). But this phenomenon is rarely found in the stomachs of smaller individuals of *S. japonicus* with a fork length less than 210 mm. Both *S. japonicus* and *S. melanostictus* are schooling, mid-to-long-distance migratory fish, with overlapping feeding grounds and migration routes. When the size disparity becomes pronounced, the smaller *S. melanostictus* is preferentially preyed upon by the larger *S. japonicus*. Specifically, when the fork length difference exceeds 60 mm, a predator–prey relationship is established, particularly in *S. japonicus* individuals exceeding 220 mm in fork length. Moreover, *S. japonicus* exhibits a higher growth coefficient than *S. melanostictus*, and as *S. japonicus* grows, its predation pressure on *S. melanostictus* increases [41,42,49]. These findings support the optimal foraging theory, which posits that predators preferentially target larger prey, as the energy gained from consuming larger individuals outweighs the energy expended in capturing them, thereby maximizing net energy gain [50,51]. Furthermore, during the overwintering period, copepods, shrimp, and other prey serve as a shared energy source for both *S. japonicus* and *S. melanostictus*. This reduces the competitive pressure between the two species for common food resources, resulting in an expansion of the suitable habitat range for *S. japonicus* at the expense of *S. melanostictus*. Consequently, the suitable habitat for *S. melanostictus* is compressed, exhibiting seasonal fluctuations characterized by periodic expansions and contractions of habitat availability [20,38]. Overall, the predator–prey dynamics between *S. japonicus* and *S. melanostictus*, driven by substantial individual size differences, modulate their competitive interactions, ultimately influencing the temporal fluctuations in their habitat ranges.

### 4.3. Correlation Between Habitat Changes of Sardinops melanostictus and S. japonicus

According to the habitat correlation analysis conducted in this study from June to November 2021, there is a clear positive correlation between the habitat changes of *S. melanostictus* and *S. japonicus* (Figure 5). Typically, interspecies competition among two or more species for limited resources such as food, space, or water is expected to result in a negative correlation, where an increase in one species is often accompanied by a decrease in another. This contradicts the conclusion drawn in this study, which suggests a tendency towards positive correlation in the habitat changes of *S. melanostictus* and *S. japonicus*. There are four primary reasons for this speculation: (1) Spatial distribution and ecological niche differentiation. *S. melanostictus* and *S. japonicus*, as long-distance migratory fish species, have constrained spatial ranges. Interspecies competition likely induces spatial and ecological niche differentiation, enabling each species to occupy distinct niches within the same habitat and thereby mitigating direct competition [19,52,53]. (2) Resource allocation and adaptive evolution. Since the 1950s, *S. melanostictus* and *S. japonicus* have established defined fishing grounds in the northwest Pacific Ocean [45,54]. Simultaneously, a stable long-term feeding ground has formed in this region. Given their similar ecological behaviors, adaptive evolution in resource allocation may have fostered the development of strategies for adaptive coexistence, mitigating intense competition and facilitating their harmonious cohabitation within the same habitat [12,38]. (3) Phasic feeding relationships. When a larger proportion of larger individuals exists within the *S. japonicus* population, they intensify predation on *S. melanostictus*. Consequently, fluctuations in the population size of *S. melanostictus* can also impact the habitat dynamics of *S. japonicus* in response [8]. (4) Complex dynamics of communities. In theory, interspecies interactions can manifest in two distinct forms: higher-order interactions and intransitive competition. The former involves changing competition coefficients, while the latter regulates competition solely through density adjustments [55]. In the primary economic fishing grounds of the northwest Pacific Ocean, prominent pelagic species include *Cololabis saira*, *S. japonicus*, *Engraulis japonicus*, and *Ommastrephes bartramii* [56]. The interplay among these species can influence competition coefficients and population densities within the biological community [2,3,57,58,59], potentially resulting in correlated habitat changes for *S. melanostictus* and *S. japonicus* due to the presence of other species. Overall, the spatial distribution and ecological niche differentiation, resource allocation and adaptive evolution, phasic feeding relationships, and complex dynamics of the community likely result in a positive correlation in the habitat changes of *S. melanostictus* and *S. japonicus*.

## 5. Conclusions

This study constructs a dynamic model of interspecies habitat association based on the competitive relationship between *S. melanostictus* and *S. japonicus*. We analyze the correlation between their habitat changes and investigate the impact of interspecies competition on the distributional differences of their habitats. Our findings indicate that interspecies competition significantly influences the habitat dynamics of *S. melanostictus* and *S. japonicus*, strengthening the correlation between their habitat variations and leading to a positive relationship between the habitat changes of the two species. Meanwhile, the icSDM developed in this study yields habitat predictions for *S. melanostictus* and *S. japonicus* that more closely align with the ecological patterns of optimal habitat distribution compared to those generated by the ssSDM. It effectively mitigates potential habitat fragmentation caused by model predictions and significantly enhances prediction accuracy. The species examined in this study, *S. melanostictus* and *S. japonicus*, exhibit significant overlap in their distribution and have relatively similar ecological habits. Environmental fluctuations can induce variations in interspecies interactions and are the primary drivers of habitat changes. The common environmental factors may attenuate the influence of interspecies relationships on habitat distribution, introducing a degree of stochasticity to the study’s findings. Additionally, the biological interactions between different age groups of *S. japonicus* and *S. melanostictus* differ; smaller and medium-sized individuals primarily compete, while larger individuals engage in both predation and competition. Limitations in fisheries data prevent precise differentiation of these specific interspecies relationships, which introduces certain constraints to this study’s conclusions.

Therefore, future studies should incorporate individual-level data—such as stomach content composition, age classes, and the degree of spatial overlap in feeding grounds—of *S. melanostictus* and *S. japonicus* to further refine our understanding of interspecific relationships across different life stages. Assigning ecologically meaningful weights to competitive, predatory, and prey interactions will improve the realism of species interaction modeling. By integrating insights from fisheries oceanography and population ecology, the development of advanced Joint Species Distribution Models (JSDMs) will allow for a more comprehensive interpretation of habitat variation among *sympatrically* distributed species. This will ultimately provide a robust scientific basis for the sustainable exploitation and management of *S. melanostictus* and *S. japonicus* populations.

## Figures and Tables

**Table 1:** Fisheries data sample size used for constructing isSDMs during June to November from 2017 to 2021.

Years	Species	Samples						
		June	July	August	September	October	November	Total
2017	*S. melanostictus*	164	177	172	157	150	132	**952**
	*S. japonicus*	124	136	106	87	88	78	**619**
	**Overlap**	**112**	**124**	**84**	**87**	**85**	**72**	**564**
2018	*S. melanostictus*	65	67	32	32	28	24	**248**
	*S. japonicus*	75	77	40	41	33	88	**354**
	**Overlap**	**65**	**67**	**30**	**31**	**28**	**24**	**245**
2019	*S. melanostictus*	43	27	30	13	13	25	**151**
	*S. japonicus*	42	27	32	13	13	26	**153**
	**Overlap**	**42**	**27**	**29**	**9**	**13**	**25**	**145**
2020	*S. melanostictus*	50	51	43	48	41	48	**281**
	*S. japonicus*	37	45	34	31	37	48	**232**
	**Overlap**	**32**	**45**	**34**	**30**	**37**	**46**	**224**
2021	*S. melanostictus*	110	131	113	125	91	83	**653**
	*S. japonicus*	70	52	32	29	42	47	**272**
	**Overlap**	**70**	**52**	**30**	**29**	**40**	**45**	**266**

Note: The samples are the sum of the number of operation sites in each month from 2017 to 2021. “Overlap” refers to the total number of sites where both *S. melanostictus* and *S. japonicus* catches occur.

**Table 2:** Description of environmental predictors and associated data sources for isSDMs model development.

Variable (unit)	Name	Time Frame	Spatial Resolution	Source	Data Acquisition
T_0 m (°C)	Sea surface temperature	June to November 2017–2021	0.25°	APDRC	http://apdrc.soest.hawaii.edu/las_ofes/v6/dataset?catitem=71 (accessed on 9 October 2023)
T_50 m (°C)	Sea 50 m depth temperature				
T_100 m (°C)	Sea 100 m depth temperature				
T_150 m (°C)	Sea 150 m depth temperature				
T_200 m (°C)	Sea 200 m depth temperature				
SSH (m)	Sea surface height				
SSS (‰)	Sea surface salinity			CMEMS	https://marine.copernicus.eu (accessed on 10 October 2023)
*Chl.*a (mg m^−3^)	Chlorophyll a				
U (m s^−1^)	X-direction				
V (m s^−1^)	Y-direction				
EKE (m s^−1^)	Eddy kinetic energy				
SSTA	Sea surface temperature anomaly			NOAA	https://origin.cpc.ncep.noaa.gov/products/analysis_monitoring/ensostuff/ONI_v5.php (accessed on 10 October 2023)
ONI	Ocean Nino index				

## Data Availability

All fisheries data supporting the findings of this study are included in this manuscript. However, these data are used under license from the Distant Squid Fisheries Sci-Tech Group (SHOU) and the East China Sea Fisheries Research Institute, and usage is only permitted by the authors, Distant Squid Fisheries Sci-Tech Group (SHOU), and the East China Sea Fisheries Research Institute upon reasonable request. All environmental data in this study are available from APDRC (http://apdrc.soest.hawaii.edu/las_ofes/v6/dataset?catitem=71 (accessed on accessed on 9 October 2023)), CMEMS (https://marine.copernicus.eu (accessed on 10 October 2023)), NOAA (https://origin.cpc.ncep.noaa.gov/products/analysis_monitoring/ensostuff/ONI_v5.php (accessed on 10 October 2023)).
